# Sustained AMPK Activation and Proline Metabolism Play Critical Roles in the Survival of Matrix-Deprived Transformed Cells

**DOI:** 10.3389/fcell.2021.771366

**Published:** 2021-11-15

**Authors:** Manipa Saha, Neha Deshpande, Abhinav Dubey, Debnath Pal, Hanudatta S. Atreya, Annapoorni Rangarajan

**Affiliations:** ^1^ Molecular Reproduction, Development and Genetics, Indian Institute of Science, Bangalore, India; ^2^ IISc Mathematics Initiative, Indian Institute of Science, Bangalore, India; ^3^ NMR Research Centre, Indian Institute of Science, Bangalore, India; ^4^ Department of Computational and Data Sciences, Indian Institute of Science, Bangalore, India; ^5^ Solid State and Structural Chemistry Unit, Indian Institute of Science, Bangalore, India

**Keywords:** AMP-activated protein kinase (AMPK), anoikis, anoikis-resistance, matrix-deprivation, proline oxidase (POX), proline metabolism, metastasis

## Abstract

Attachment to the matrix is critical for the survival of adherent cells, whereas detachment triggers death by apoptosis. Therefore, solid tumors must acquire the ability to survive the stress of matrix-detachment to transit through circulation and seed metastases. Although a central role for energy metabolism in cancer progression is well established, what distinguishes its role in the cellular state of the matrix-deprived form compared to the matrix-attached form is not fully understood yet. Using an *in vitro* transformation model dependent on simian virus 40 (SV40) small t (ST) antigen for cellular survival and proliferation in matrix-deprived conditions, we demonstrate that 5′-adenosine monophosphate-activated protein kinase (AMPK) activity is elevated and sustained under matrix-deprived conditions in ST-expressing fibroblasts. Additionally, these cells display elevated energy (ATP) levels under matrix-deprived conditions in contrast to cells lacking ST expression. The elevated ATP levels are coupled to increased levels of proline in ST-expressing cells, as revealed by metabolomics studies. The AMPK-dependent upregulation of proline oxidase, an enzyme of proline degradation, is a key link for elevated ATP levels. This functional link is further established by proline supplementation concomitant with AMPK activation in matrix-deprived cells lacking ST antigen, yielding ATP and enhancing survival. Thus, our data establishes a key role for AMPK-dependent regulation of proline metabolism in mediating energy homeostasis and promoting survival of matrix-deprived cells. These findings identify key markers that distinguish the metabolic states of matrix-detached and matrix-attached transformed cells and have implications in developing novel therapeutic strategies for specifically targeting matrix-detached metastasizing cancer cells.

## 1 Introduction

The spread of cancer by metastasis is the major cause of cancer-associated death. Advancement of cancer from the formation of a primary tumor to the seeding of metastases comprises a complex series of events that encompass multiple hurdles to be overcome by cancer cells (Mehlen and Puisieux, 2006). It is increasingly being recognized that metabolic adaptation is a key attribute of cancer cells that enables them to withstand adverse conditions during the course of cancer progression (Hanahan and Weinberg, 2011). A fundamental requirement of cancer cells to seed metastases is the ability to withstand the stress of matrix-deprivation. It is known that normal or untransformed adherent cells, such as epithelial cells, endothelial cells and fibroblasts, undergo apoptosis upon cell-detachment from the matrix, which has been termed as “anoikis” (Frisch and Francis, 1994). In contrast, transformed cancer cells evade anoikis and survive under matrix-deprived conditions, thus attaining anchorage-independence (Paoli et al., 2013). This, in turn, is an important hallmark property of cancer cells that endows them with the ability to survive in circulation and seed metastases at distant locations (Mehlen and Puisieux, 2006). Therefore, a better understanding of the mechanisms that enable cancer cells to survive the stress of matrix-deprivation can help in identifying therapeutic strategies to prevent metastasis.

**GRAPHICAL ABSTRACT:**
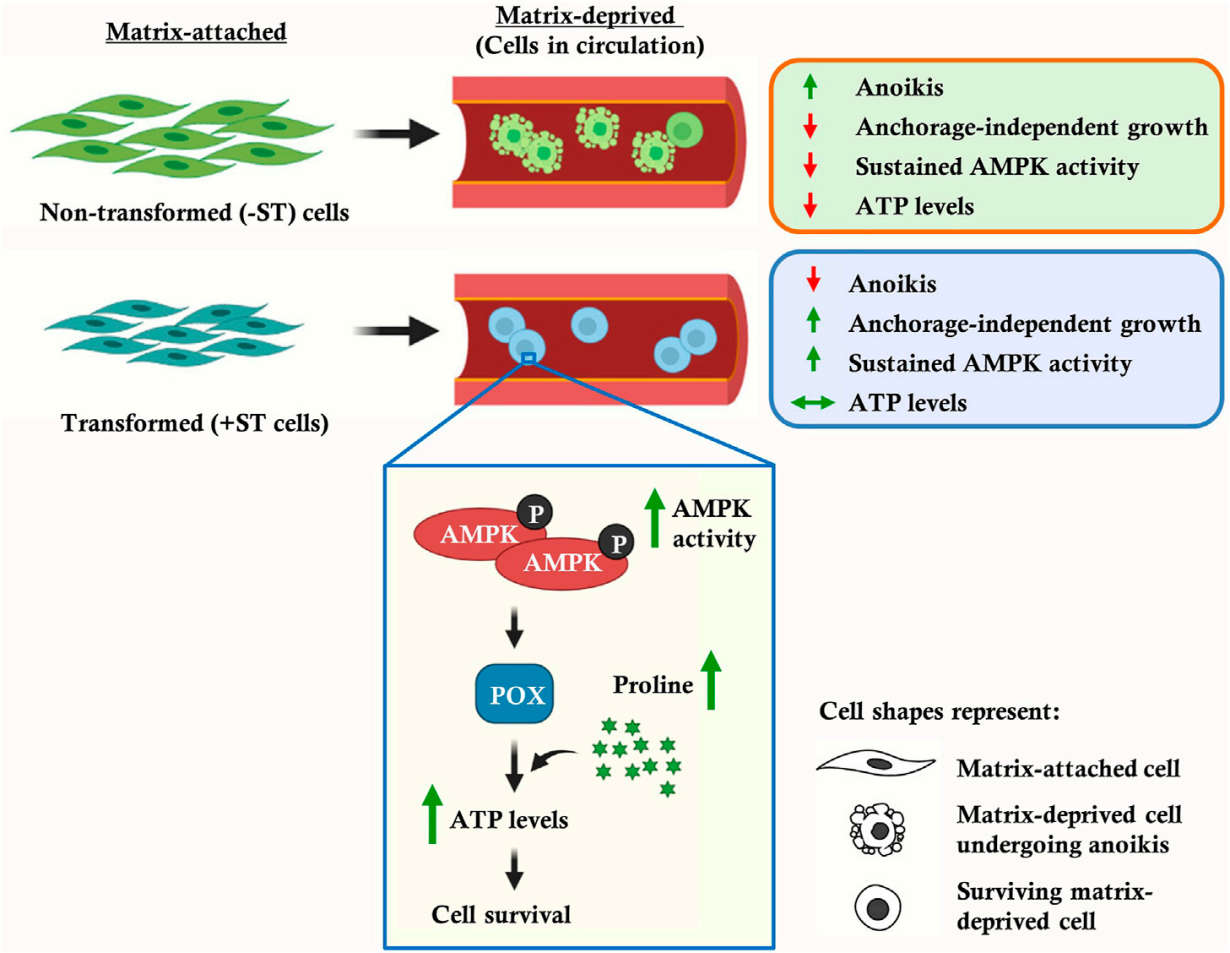


Tumor cells employ different strategies to overcome anoikis (Guadamillas et al., 2011), chief amongst them being mechanisms that prevent apoptosis which is triggered upon matrix-deprivation. For example, Akt has been shown to phosphorylate and thus inhibit pro-apoptotic proteins such as BAD, caspase-9 and GSK3β; the transcriptional co-repressor CTBP1 has been shown to regulate the expression of pro-apoptotic proteins Bax and p21; and the transcription factor Twist is known to regulate the expression of anti-apoptotic protein Bcl-2 and pro-apoptotic protein Bax (Guadamillas et al., 2011). Apart from inhibition of apoptosis, epithelial-mesenchymal transition (EMT), the phenomenon of entosis, cell aggregation, and autophagy have been identified as strategies that enable tumor cell survival under the stress of detachment (Guadamillas et al., 2011). Other studies have shown that detached cells are bioenergetically compromised (Schafer et al., 2009), suggesting the need for metabolic adaptations to bypass this state. Yet, metabolic alterations in matrix-deprived states of cancer cells remain poorly understood.

The 5′-adenosine monophosphate-activated protein kinase (AMPK) is a central metabolic regulator that is primarily activated upon energy depletion in cells (Hardie et al., 2012). It is a heterotrimeric protein consisting of a catalytic *α* subunit (encoded by *α*1 and *α*2), a scaffolding *β* subunit (encoded by *β*1 and *β*2) and a regulatory *γ* subunit (encoded by *γ*1, *γ*2, and *γ*3). Under conditions of low cellular energy, binding of AMP leads to allosteric activation of AMPK by promoting phosphorylation at T172 residue by upstream kinases as well as by preventing dephosphorylation (Hardie, 2015). AMPK has been shown to play a protective role under various stress conditions like nutrient-deprivation, endoplasmic reticulum stress, oxidative stress and hypoxia (Viollet et al., 2010). Recent data from our laboratory as well as from others have shown a critical requirement for AMPK in anoikis-resistance and anchorage-independent growth (Jeon et al., 2012; Ng et al., 2012; Hindupur et al., 2014; Sundararaman et al., 2016; Saha et al., 2018). AMPK has been implicated in anoikis-resistance through regulation of NADPH homeostasis and inhibition of protein synthesis (Jeon et al., 2012; Ng et al., 2012). These data suggest that AMPK might bring about energy homeostasis of matrix-deprived cells. Nevertheless, a role for AMPK in metabolic reprogramming in the matrix-deprived state is not fully understood.

In this study, using *in vitro* transformed human fibroblasts that are dependent on the simian virus 40 (SV40) ST antigen for their matrix-deprived survival and growth (Rangarajan et al., 2004), we show that the presence of ST antigen helps sustain AMPK activity in matrix-deprived cells, which in turn helps maintain ATP levels. We further show that the elevated ATP level in these cells is coupled to increased levels of proline, as revealed by NMR-based metabolomics studies. We identify AMPK-dependent upregulation of proline oxidase, a key enzyme of proline catabolism, as an important link for elevated ATP levels in matrix-deprived cells. Thus, our data establishes a key role for AMPK-dependent regulation of proline metabolism in regulating energy homeostasis in matrix-deprived state. These findings identify metabolic reprogramming of tumor cells during cancer progression, and suggest targeting proline metabolism as a novel therapeutic strategy to prevent cancer metastasis.

## 2 Results

### 2.1 Matrix-Deprived +ST Cells Show Enhanced Viability

**FIGURE 1:**
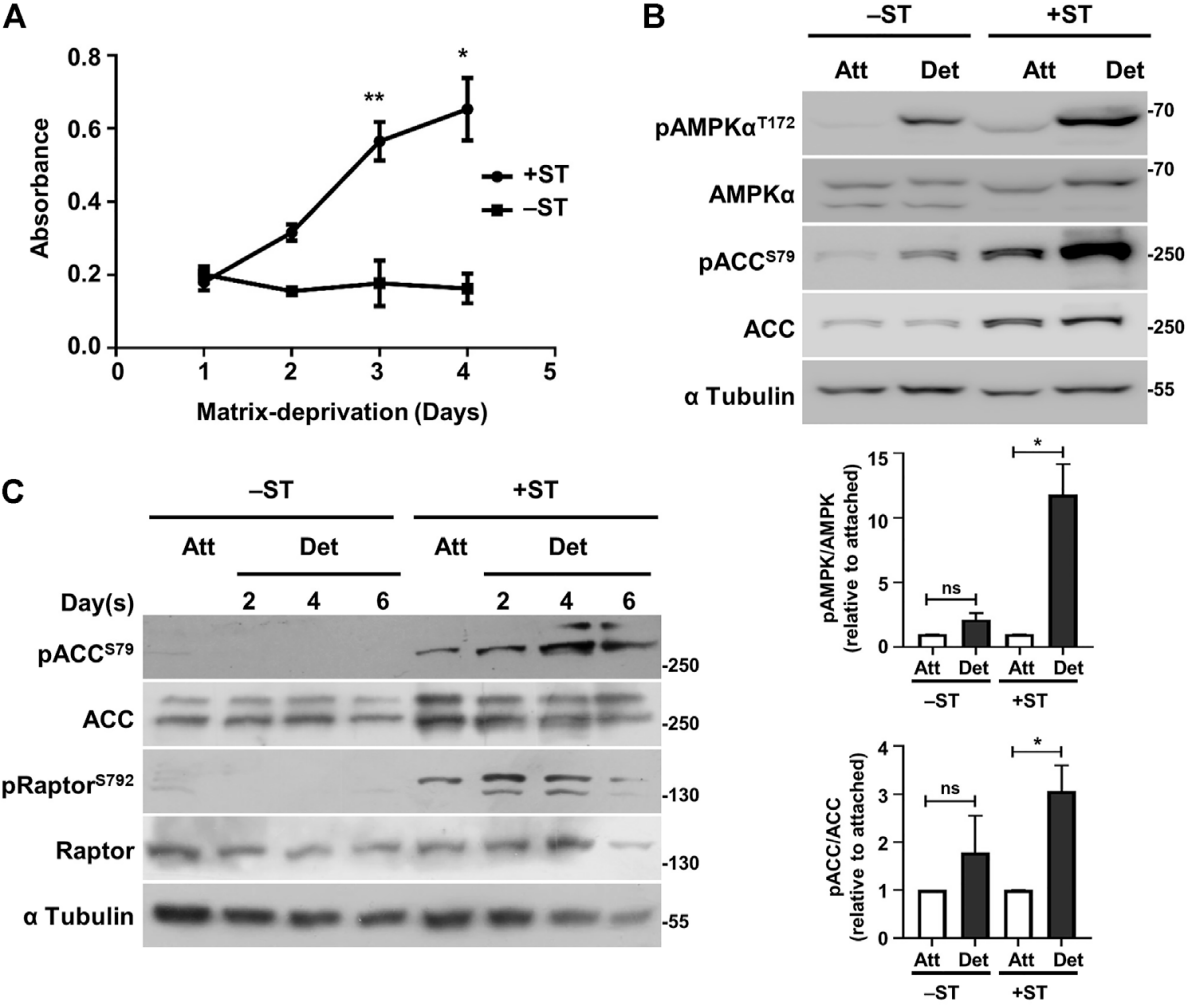
+ST cells display increased survival and sustained AMPK activity under matrix-deprived conditions as compared to −ST cells: **(A)** −ST and +ST cells grown in a 96-well ultra-low attachment plate that prevents cell-attachment were subjected to MTT assay at specified time periods. Absorbance of formazan crystals dissolved in DMSO was monitored for 4 days; *n* = 4. Error bars represent mean ± SEM. **(B)** −ST and +ST cells subjected to conditions of attachment (Att) and matrix-deprivation (Det) for 24 h, were harvested for immunoblot analyses for the specified proteins. Graphs represent densitometric quantification of phospho-proteins to total proteins normalized to *α*-tubulin and relative to the attached condition for each cell line; error bars represent mean ± SEM; *n* = 3. **(C)** −ST and +ST cells subjected to matrix-deprivation (Det) for 2, 4 and 6 days were harvested for immunoblot analyses for the specified proteins. Adherent/attached cells (Att) were used as controls; *n* = 3.

Human fibroblasts expressing SV40 LT antigen, the catalytic subunit of human telomerase (hTERT), oncogenic H-Ras and additionally the ST antigen of SV40 (henceforth referred to as plus ST or +ST cells) form anchorage-independent colonies as opposed to those not expressing the ST antigen (henceforth referred to as minus ST or –ST cells) (Rangarajan et al., 2004). Anchorage-independent growth requires the ability to survive as well as proliferate under matrix-deprived conditions. We, therefore, gauged the viability and proliferation of matrix-detached +ST and −ST cells. To do so, we seeded both the cell types in a 96-well ultra-low attachment plate (that prevents adhesion) and monitored their viability using an MTT assay. We observed that while +ST cells showed an increase in absorbance under matrix-deprived conditions, indicating cell survival and proliferation, −ST cells failed to do so (Figure 1A). These results suggested a role for ST antigen in the survival and proliferation of matrix-deprived cells.

### 2.2 Sustained AMPK Activity in Matrix-Deprived +ST Cells

We probed mechanistically into how ST antigen promotes survival of matrix-deprived cells. Earlier work from our laboratory demonstrated that ST antigen-mediated PP2A inhibition results in sustained AMPK activation under the stress of glucose-deprivation (Kumar and Rangarajan, 2009). More recent work from our laboratory has revealed AMPK activation in matrix-deprived cells, and identified its critical role in anchorage-independent survival and growth (Hindupur et al., 2014; Sundararaman et al., 2016; Saha et al., 2018). Similarly, studies from other laboratories have also identified functions of AMPK in anoikis-resistance (Jeon et al., 2012; Ng et al., 2012; Avivar-Valderas et al., 2013). We, therefore, investigated the role of AMPK in ST antigen-mediated survival of matrix-deprived tumor cells.

In order to test the activation status of AMPK under matrix-deprived conditions, −ST and +ST cells grown in adherent conditions or under matrix-deprived conditions were harvested for immunoblotting. AMPK phosphorylated on the critical T172 residue signals its activated state (Hardie, 2015). We observed elevated pAMPKα^T172^ levels in +ST cells that were matrix-deprived for 24 h compared to −ST cells (Figure 1B). Moreover, a robust increase in the phosphorylated form of acetyl-CoA carboxylase (ACC), a bona fide substrate of AMPK (Davies et al., 1990), was observed in matrix-deprived +ST cells, but not in −ST cells, suggesting elevated AMPK activity in matrix-deprived +ST cells (Figure 1B).

We next investigated the status of AMPK activity when these cells were matrix-deprived for prolonged periods. With increasing time under matrix-deprived conditions, we observed AMPK read-out pACC^S79^ only in +ST cells (Figure 1C). We obtained similar data with yet another read-out of AMPK, Raptor (Gwinn et al., 2008) (Figure 1C), suggesting that only +ST cells are capable of sustaining AMPK activity long term. Thus, these data suggested that the presence of ST antigen results in sustained AMPK activation upon matrix-deprivation. ST antigen is known to inhibit PP2A (Pallas et al., 1990), a cellular phosphatase that inhibits AMPK (Joseph et al., 2015; Cairns et al., 2020), and could thus contribute to elevated AMPK activity.

### 2.3 AMPK Regulates Survival of Matrix-Deprived +ST Cells

**FIGURE 2:**
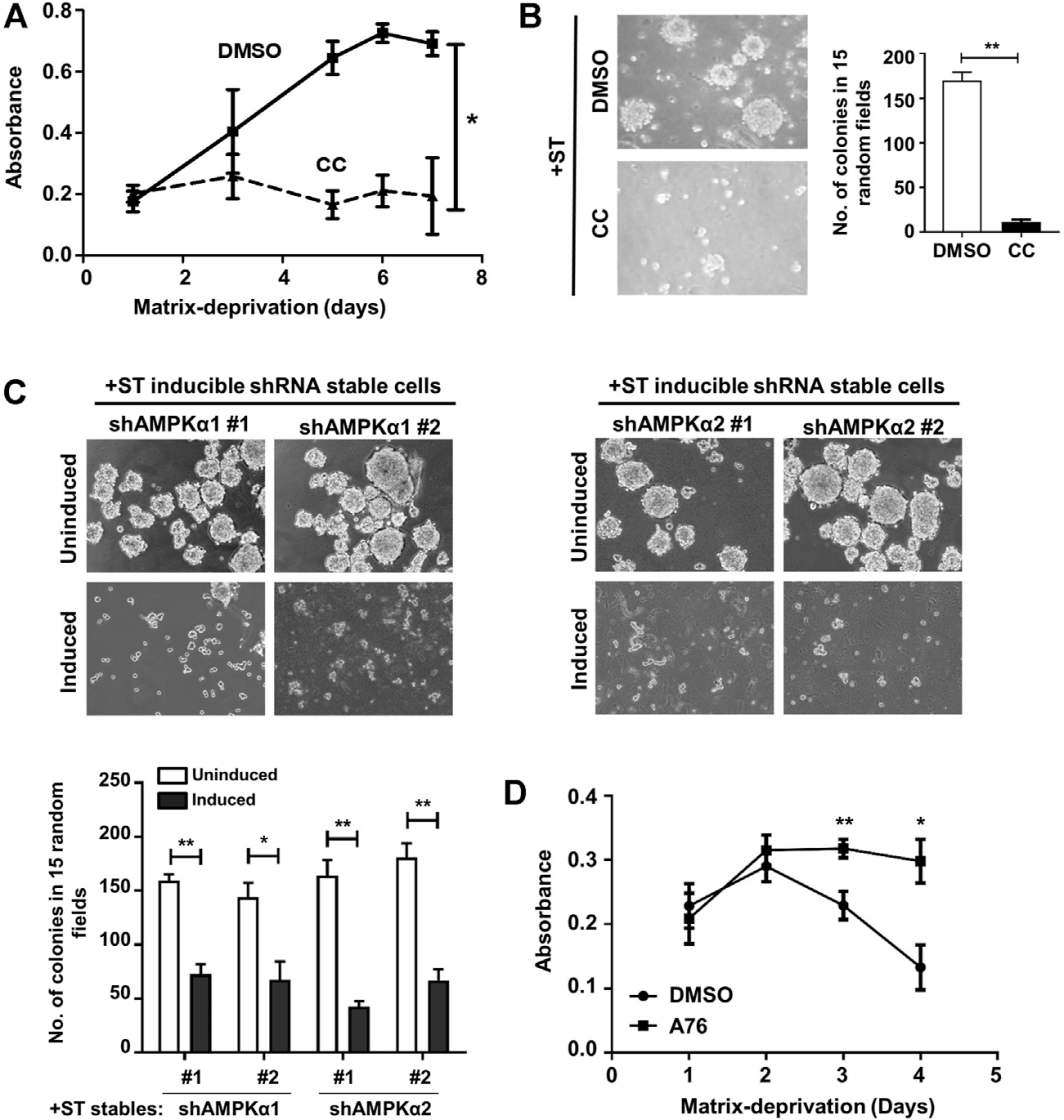
AMPK activation regulates survival and proliferation of ST expressing cells: **(A)** Matrix-deprived (Det) +ST cells treated with either vehicle control (DMSO) or AMPK inhibitor, compound C (CC; 10 μM), for 24 h were cultured in a 96-well ultra-low attachment plate that prevents cell-attachment and subjected to MTT assay at specified time periods. Absorbance was monitored for 7 days; *n* = 4. Error bars represent mean ± SEM. **(B)** Cells as treated in **(A)** were subjected to colony formation in methyl cellulose. Graph represents the number of colonies in 15 random fields, counted after 15 days; *n* = 3. **(C)** +ST cells stably expressing specified shRNA under Tet-on inducible promoter were subjected to colony formation assay in methyl cellulose. Uninduced cells were used as control; 5 µg/ml doxycycline was used for induction. Graph represents the number of colonies in 15 random fields, counted after 15 days; *n* = 3. **(D)** Matrix-deprived (Det) −ST cells treated with either vehicle control (DMSO) or AMPK activator, A-769662 (A76; 150 μM), were subjected to MTT assay in a 96-well ultra-low attachment plate. Absorbance was monitored for 4 days; *n* = 4. Error bars represent mean ± SEM.

We next investigated if sustained AMPK activity was responsible for the survival and proliferation of matrix-deprived +ST cells. To do so, we inhibited AMPK in +ST cells using a pharmacological agent, compound C (Zhou et al., 2001). The inhibitor was found to be efficient at a concentration of 10 µM, as revealed by a reduction in the levels of pACC^S79^; the expression of total ACC remained unchanged (Supplementary Figure S1A). In order to test the effects of AMPK inhibition on cell viability and proliferation under matrix-deprived conditions, +ST cells were seeded in a 96-well ultra-low-attachment plate in the presence or absence of compound C, and MTT assay was undertaken. Our observations revealed that the survival and proliferation of +ST cells were significantly reduced under matrix-deprived conditions upon AMPK inhibition (Figure 2A). The cells also displayed compromised anchorage-independent colony formation potential in the presence of compound C as compared to DMSO-treated cells (Figure 2B).

Compound C and sunitinib are the only pharmacological inhibitors of AMPK currently available. Of these, Compound C is used extensively in literature as a bona fide AMPK inhibitor. However, it is also known to inhibit other protein kinases with equal or greater potency (Bain et al., 2007). Similarly, sunitinib shows a broad-spectrum inhibition of multiple protein kinase families (Karaman et al., 2008). Therefore, to eliminate any ambiguous interpretation of the results obtained using Compound C, we further validated our data using an inducible system for RNAi mediated depletion of the catalytic α subunits of AMPK. For this, we generated +ST cells stably expressing two independent doxycycline-inducible shRNA constructs each targeted against both the isoforms of the catalytic subunit of AMPK, *α*1 and *α*2. A key advantage of this system is the ability to use the same pool of stable cells as both the control (without induction) and test (with induction), thereby eliminating concerns pertaining to the unintentional clonal selection between “empty-vector control” and “shRNA stable” pools (Wiederschain et al., 2009). Following induction with doxycycline (5 µg/ml) for 48 h, knockdown of the respective AMPKα subunits was confirmed using qPCR (Supplementary Figure S1B) which revealed greater than 70% knockdown**.** A western blot analysis confirmed depletion of AMPK at protein levels and further showed a decrease in pACC^S79^ levels revealing abrogation of AMPK signaling upon shRNA induction (Supplementary Figure S1C; data not shown for sequence #2). When these cells were subjected to soft-agar colony formation assay, anchorage-independent colony formation capability was compromised in +ST cells depleted of AMPKα1 subunit following doxycycline induction in both sequence #1 and #2 expressing cells as compared to uninduced control cells (Figure 2C). Similar results were obtained in +ST cells stably expressing two independent shRNA sequences (seq#1 and #2) against the AMPKα2 subunit (Figure 2C). Thus, these data further strongly validated a role for AMPK in promoting the survival of matrix-deprived cells expressing ST antigen.

To address the question conversely, we enquired if AMPK activation would promote the survival of matrix-deprived −ST cells. To do so, we activated AMPK in −ST cells using a pharmacological activator, A-769662 (Cool et al., 2006; Göransson et al., 2007; Sanders et al., 2007). An increase in levels of pRaptor^S792^ confirmed AMPK activation (Supplementary Figure S1D) in A-769662-treated −ST cells under matrix-deprived conditions. Further, AMPK activation enhanced the long-term survival of matrix-deprived −ST cells (Figure 2D). Together, these data identify a key role for ST antigen-mediated sustained AMPK activity in promoting cell survival under the stress of matrix-deprivation.

### 2.4 ST Antigen Helps Maintain Energy Homeostasis

**FIGURE 3:**
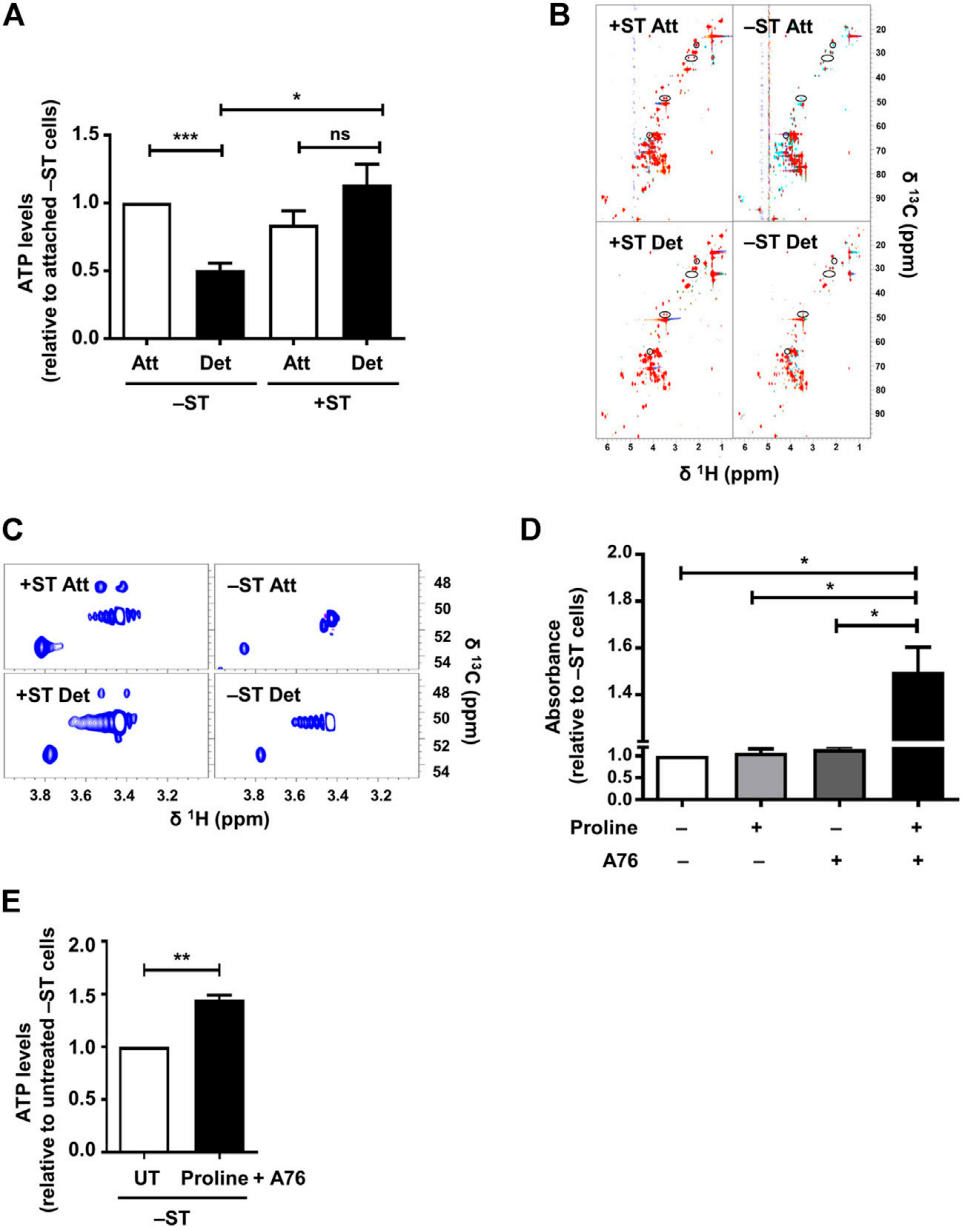
Proline supplementation in combination with AMPK activation increases ATP levels and enhances survival of −ST cells: **(A)** –ST and +ST cells subjected to conditions of attachment (Att) and detachment (Det), were harvested for the measurement of ATP levels. Graph represents ATP levels; *n* = 5. Error bars represent mean ± SEM. **(B,C)** −ST and +ST cells subjected to conditions of attachment (Att) and detachment (Det), were harvested for 2D [^13^C, ^1^H] HSQC NMR spectra. **(B)** The spectra from 5 biological repeats are overlaid. Peaks present in +ST cells but absent in −ST cells are circled. **(C)** Spectra shows zoomed-in region of two such peaks exclusively present in +ST cells. **(D)** −ST cells were cultured in a 96-well ultra-low attachment plate in the presence of proline (200 mg/L; w/v) and AMPK activator, A-769662 (A76; 150 μM), either singly or in combination and subsequently MTT assay was performed. Absorbance was monitored after 48 h; *n* = 4. Error bars represent mean ± SEM. **(E)** –ST cells subjected to matrix-deprived conditions for 48 h in the presence of proline (200 mg/L) and AMPK activator A-769662 (A76; 150 μM) were harvested for measurement of ATP levels. Graph represents ATP levels; *n* = 4. Error bars represent mean ± SEM.

We next investigated how ST antigen-mediated sustained AMPK activity contributes to tumor cell survival in matrix-deprived state. AMPK is a cellular energy sensor that is activated upon low energy conditions and functions to maintain energy homeostasis (Hardie et al., 2012) by enabling ATP production. We, therefore, checked ATP levels in −ST and +ST cells upon matrix-deprivation. The assay is based on a luciferase-catalyzed reaction that utilizes ATP and luciferin as substrates to emit light which can be recorded as luminescence. The basal levels of ATP in −ST and +ST cells grown under adherent conditions did not differ significantly (Figure 3A). However, matrix-deprived −ST cells were found to have reduced ATP levels as compared to their adherent counterparts (Figure 3A). On the other hand, in +ST cells, ATP levels between attached and detached conditions were almost similar (Figure 3A). These data suggested that +ST cells, with sustained AMPK activity, were able to maintain their ATP levels under the stress of matrix-detachment compared to −ST cells.

### 2.5 Proline Levels Are Elevated in +ST Cells

Since our results revealed a difference in ATP levels between −ST and +ST cells, and identified sustained activation of AMPK, which is known to turn on energy-producing metabolic pathways (Hardie et al., 2012), in +ST cells, we investigated the differences in metabolic pathways between these two cell types. To do so, we undertook 2D [^13^C, ^1^H] HSQC NMR experiments for metabolic profiling of +ST and −ST cells under attached and detached conditions. Analyses of the NMR spectra revealed some peaks exclusively present in +ST cells, under both matrix-attached and matrix-detached conditions. Using 2D [^13^C, ^1^H] HSQC-TOCSY, we found that these peaks belong to proline, a non-essential amino acid (Figures 3B,C and Supplementary Figure S1E). This hinted at a possible role for altered proline metabolism in +ST cells.

### 2.6 Proline Supplementation Enhances Survival of −ST Cells

Since we found elevated proline levels in +ST cells that also have sustained AMPK activation, and degradation of proline by AMPK has been shown to generate ATP under various stress conditions (Liu et al., 2012a; Elia et al., 2017), we investigated if supplementation of proline would rescue the bioenergetic defect in −ST cells and promote cell survival. Towards this, we cultured matrix-deprived −ST cells in the presence of exogenously supplemented proline, with and without AMPK activation using A-769662, and undertook MTT based cell survival assay. Interestingly, we observed that in the simultaneous presence of proline and A-769662, absorbance of −ST cells under matrix-deprived conditions was higher in comparison to parental cells or to cells in the presence of either proline or A-769662 alone (Figure 3D). Thus, AMPK activation in the presence of proline sufficed to enhance the viability of matrix-detached −ST cells. Further, these cells showed elevated levels of ATP (Figure 3E). Together, these data suggested that AMPK activation promotes energy generation *via* proline metabolism upon matrix-detachment, thereby supporting matrix-deprived cell survival.

### 2.7 AMPK Positively Regulates Proline Oxidase

**FIGURE 4:**
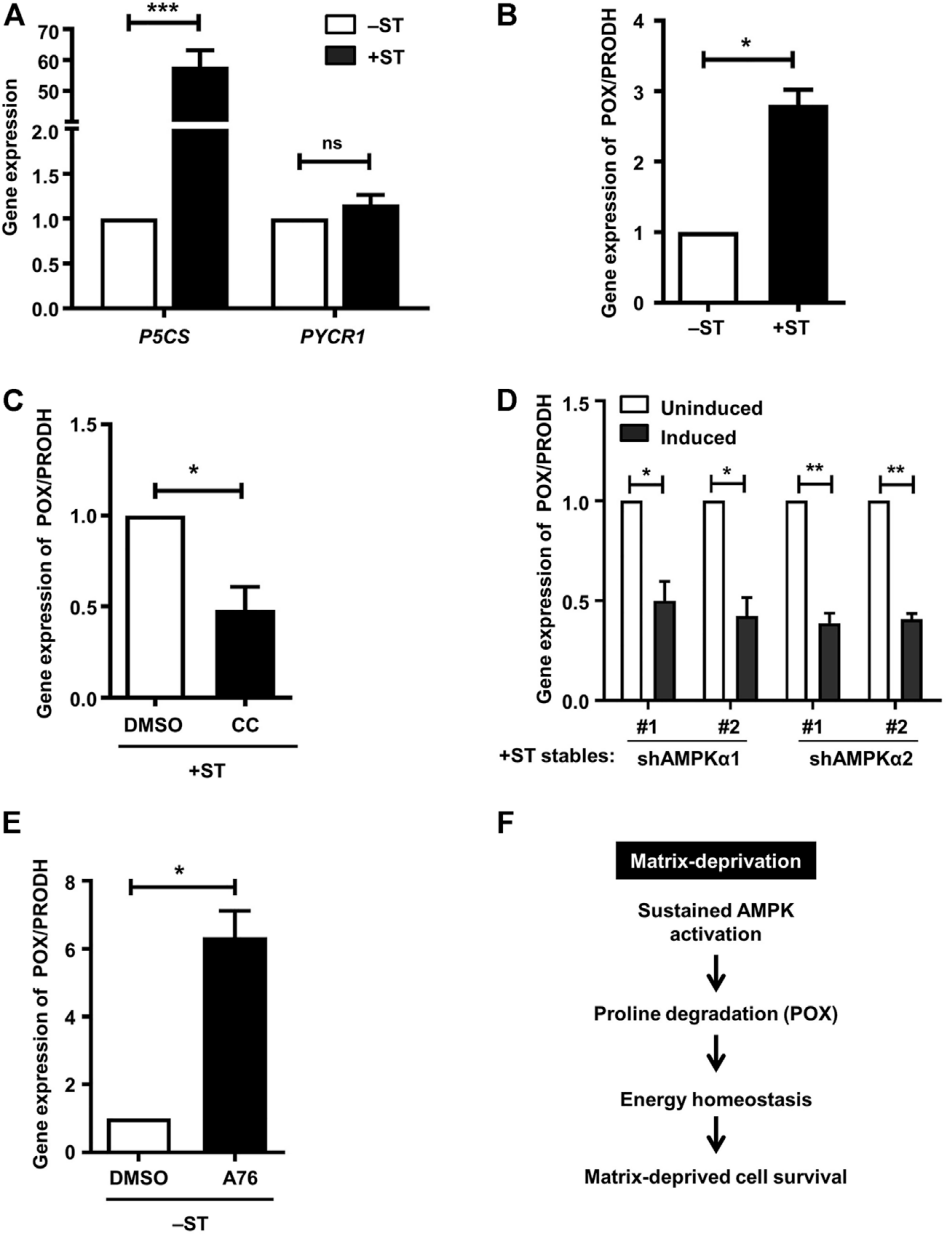
AMPK activation positively regulates proline oxidase: **(A)** Adherent −ST and +ST cells were harvested for qPCR analysis of P5CS and PYCR1. Graph represents fold change of gene expression normalized to housekeeping gene β2M. Error bars represent mean ± SEM; *n* = 4. **(B–E)** qPCR analysis was done for expression of POX/PRODH under the following conditions: **(B)** Adherent −ST and +ST cells cultured for 24 h, *n* = 3; **(C)** Adherent + ST cells treated with AMPK inhibitor compound C (CC; 10 μM) or vehicle control DMSO for 24 h; *n* = 4; **(D)** Adherent +ST cells stably expressing the specified shRNA under Tet-on inducible promoter induced with 5 µg/ml doxycycline for 48 h; uninduced cells were used as control; *n* = 3; **(E)** Adherent −ST cells treated with AMPK activator A-769662 (A76; 150 μM) or vehicle control DMSO for 24 h; *n* = 3. All graphs represent fold change of gene expression normalized to housekeeping gene β2M. Error bars represent mean ± SEM. **(F)** Sustained AMPK activation promotes energy homeostasis and matrix-deprived survival *via* regulation of POX expression and thus proline metabolism.

We investigated the mechanisms that might lead to altered proline metabolism in +ST cells. We first investigated the enzymes that are involved in proline biosynthesis. We undertook RT-PCR based experiments to check the transcript levels of P5CS/ALDH18A1 and PYCR1 that are involved in the biosynthesis of proline from glutamine. We failed to observe any changes in the transcript levels of PYCR1 between −ST and +ST cells (Figure 4A); however, we saw ∼50-fold increase in the transcript levels of P5CS in +ST cells compared to −ST cells (Figure 4A), suggesting its possible involvement in proline biosynthesis and accumulation in +ST cells. We next investigated the enzyme involved in proline catabolism, proline dehydrogenase (PRODH), also known as proline oxidase (POX). Under conditions of stress, AMPK has been shown to generate ATP by proline degradation through upregulating proline oxidase (POX) (Liu et al., 2012a; Elia et al., 2017). We, therefore, checked the transcript levels of POX/PRODH in −ST and +ST cells. We observed a ∼3-fold increase in POX transcripts in +ST cells as compared to −ST cells (Figure 4B). Thus, these data suggested that both enzymes chiefly involved in the proline biosynthesis pathway (P5CS) and proline degradation pathway that generates energy (POX) are elevated in +ST cells.

We tested whether transcript levels of P5CS and POX are regulated by AMPK in these fibroblast cell lines. Towards this, we inhibited AMPK in +ST cells using compound C. The presence of AMPK inhibitor failed to alter the levels of P5CS in +ST cells (Supplementary Figure S1F), suggesting that proline biosynthesis in these cells is not mediated by sustained AMPK activation. On the other hand, as shown in Figure 4C, we observed that AMPK inhibition led to a significant reduction in POX transcript levels. We further corroborated this result using +ST cells stably expressing two different sequences each of inducible shRNAs against AMPKα1 or AMPKα2. Induction of shRNA led to a reduction in POX transcript levels as compared to uninduced control cells (Figure 4D). Further, AMPK activation in −ST cells using A-769662 resulted in increased transcript levels of POX (Figure 4E). Together, these data identified that energy (ATP) restoration in matrix-detached transformed cells is mediated *via* AMPK-dependent proline degradation achieved through the upregulation of POX.

## 3 Discussion

Much of our current understanding of the fundamental molecular pathways perturbed during cancer development stems from the study of transforming viruses and the oncoproteins they encode. For instance, SV40 LT antigen and human papilloma virus oncoproteins E6 and E7 have helped understand the tumor-preventing roles of p53 and pRb, two important cellular tumor suppressors that are often mutated in cancer (Chellappan et al., 1992; Ali and DeCaprio, 2001; Rangarajan et al., 2004). Furthermore, introduction of these viral oncogenes into normal cells have allowed the development of step-wise *in vitro* models of carcinogenesis that serve as robust tools in identifying critical molecular perturbations required for cancer development and progression.

One such *in vitro* transformation model is the +ST cell line generated from primary human foreskin fibroblasts that have been transformed through the serial introduction of simian virus 40 early region (coding for the large T and small t antigen), the catalytic subunit of human telomerase (hTERT), and an oncogenic allele of H-Ras (H-Ras^V12^) (Rangarajan et al., 2004). The transformed +ST cells have a counterpart in −ST cells which lack the SV40 ST antigen but express the rest of the genetic elements. In contrast to the tumorigenic +ST cells, −ST cells are non-tumorigenic (Hahn et al., 2002; Rangarajan et al., 2004), highlighting an important role for ST antigen in human cell transformation. Moreover, unlike cancer cell lines, these cells do not exhibit genomic instability (Hahn et al., 2002). Thus, the two cell lines differing in the expression of only one genetic element, the SV40 ST antigen, serve as matched controls allowing for an accurate comparison to delineate the critical molecular pathways required for cancer cell survival and progression.

Previous work from our laboratory has identified that ST antigen-mediated AMPK activation under glucose-deprivation is required for maintaining energy homeostasis by inhibiting protein synthesis and inducing autophagy (Kumar and Rangarajan, 2009). This suggested a critical role of ST antigen in mitigating nutrient-deprivation stress experienced by incipient tumours. Matrix-deprivation is yet another stress experienced by cancer cells during transit from the primary tumour to distant sites. While transformed cells harbouring SV40 ST antigen are known to be independent of the requirement of matrix-adhesion, perturbation of the cellular machinery that enables ST-dependent survival in matrix-deprived states is not known. In an attempt to understand this, in this study, we have compared cells expressing ST antigen (+ST cells) with cells which do not express ST antigen (−ST cells) under conditions of matrix attachment and detachment.

The Akt/PKB pathway is majorly involved in cell survival (Song et al., 2005). Previous work from our laboratory and others demonstrated that activation of AMPK under conditions of matrix-deprivation stress is required for cell survival *via* the inhibition of apoptosis (Hindupur et al., 2014; Saha et al., 2018) and protein synthesis (Ng et al., 2012). We further showed an inverse relationship between AMPK and Akt in matrix-deprivation (Saha et al., 2018). In this manuscript, we revealed that while matrix-deprived +ST cells have sustained AMPK activation, −ST cells failed to display this. Further, we show that −ST cells have compromised survival under matrix-deprived conditions, as opposed to +ST cells. A possible role for AMPK is suggested by the observation that AMPK inhibition in +ST cells compromises cell survival in detachment, while AMPK activation in −ST cells increases cell survival, at least partly. Moreover, as previously observed in other cell lines (Ng et al., 2012; Saha et al., 2018), inhibition of AMPK in matrix-deprived +ST cells led to an increase in Akt and mTOR signaling (data not shown). These results suggest that cell survival under the stress of matrix-deprivation is primarily dependent on AMPK signaling. Further, we show that while matrix-deprived +ST cells maintain their ATP levels comparable to adherent cells, matrix-deprived −ST cells fail to do so. This, we believe, is because of the ability of +ST cells to activate AMPK, as it functions within a cell to restore energy homeostasis.

Our results demonstrated that while matrix-deprived +ST cells are capable of sustained activation of AMPK, −ST cells fail to do so. We have previously shown that matrix-deprivation leads to AMPK activation by increasing ROS and calcium (Sundararaman et al., 2016); thus, irrespective of ST antigen, cells can activate AMPK under matrix-deprivation. While ST-expressing cells continue to show elevated pAMPK after 24 h, in the absence of ST, this is not the case. ST antigen is known to mediate its cellular effects by interacting with and inhibiting PP2A (Pallas et al., 1990), which is also known to dephosphorylate AMPK (Joseph et al., 2015; Cairns et al., 2020). Hence, we speculate that the absence of ST antigen allows for the dephosphorylation of AMPK in matrix-deprived −ST cells. In contrast, the presence of ST antigen sustains long term AMPK activation in matrix-deprived +ST cells.

Previous results from our laboratory have identified important roles for AMPK in mediating cell survival of matrix deprived breast cancer cells through the inhibition of apoptosis and protein synthesis and the alteration of cellular metabolism (Hindupur et al., 2014; Saha et al., 2018). Likewise, activated AMPK enhances matrix-deprived cell survival by maintaining energy homeostasis through the inhibition of mTORC1 and protein synthesis in H-Ras-transformed MEFs (Ng et al., 2012). However, even though AMPK is a master regulator of metabolism, the metabolic profiles of matrix-attached and matrix-deprived cancer cells remain relatively unexplored. Using NMR-based metabolomics studies, we identified that, under matrix deprivation, +ST cells have increased intracellular proline level which is coupled to their elevated levels of ATP compared to −ST cells. The activated AMPK in matrix-deprived +ST cells leads to the upregulation of proline oxidase (POX) which, in turn, facilitates the utilization of proline as an energy source to maintain ATP levels. Further, complementation of proline along with AMPK activation improved survival of matrix-deprived −ST cells, suggesting that proline catabolism can serve as an energy-generating pathway during matrix-deprivation. Hahn et al. (2002) had previously shown that −ST cells are dependent on glutamine for their survival and proliferation in adherent cultures, whereas +ST cells could proliferate even under conditions of glutamine-deprivation. Our current study has identified yet another amino-acid (proline) metabolic pathway utilized differently by ST-expressing cells.

A role for proline catabolism was previously reported in the survival of breast cancer cells grown as 3D spheroids (Elia et al., 2017). In contrast to our results, they observed decreased intracellular proline levels upon matrix-deprivation. Thus, what leads to the accumulation of proline in +ST cells needs to be explored. Proline biosynthesis from glutamate is catalyzed by two enzymes—pyrroline-5-carboxylate synthase (P5CS) synthesizes the intermediate pyrroline-5-carboxylate (P5C) from glutamate, which is then converted to proline by P5C reductase (PYCR/P5CR). We detected elevated expression of P5CS in +ST cells, while no significant change was observed in PYCR1 expression. However, PC5S expression was found to be AMPK-independent. c-myc is another protein that is known to be stabilized by ST antigen (Klucky and Wintersberger, 2007), and c-myc is known to upregulate P5CS (Liu et al., 2012b). Thus, it is possible that while ST-antigen mediated stabilization of c-myc promotes proline biosynthesis, ST-antigen mediated sustained AMPK activation promotes proline catabolism to generate energy under matrix-deprived condition. In another oncovirus-infected model of carcinogenesis, Kaposi’s sarcoma-associated herpesvirus (KSHV)-infected endothelial and breast epithelial cells relied on proline metabolism for growth in 3D culture (Choi et al., 2020). Similar to our results, the authors reported increased intracellular proline accumulation in KSHV-infected cells, resulting from increased P5CS enzyme activity caused by direct binding of the KSHV K1 oncoprotein to P5CS (Choi et al., 2020). Thus, data from several different cell line models demonstrate that cancer cells rely predominantly on proline metabolism under conditions of matrix-deprivation.

Our data point towards a key role for proline metabolism in aiding cell survival during matrix-deprivation, thereby promoting cancer metastasis. This is in keeping with recent reports demonstrating the contribution of proline metabolism in several cancer cell properties such as proliferation, migration, inhibition of apoptosis and induction of autophagy (Liu et al., 2012a; Ding et al., 2017; Ye et al., 2018; Fang et al., 2019; Zhuang et al., 2019; Ding et al., 2020; Liu et al., 2020). Thus, apart from matrix-deprived cell survival, proline metabolism is involved in other hallmark properties of cancer as well as in the evasion of various stress conditions. Based on our observations, we propose that ST antigen-mediated AMPK activation upregulates proline-degrading enzyme POX, leading to ATP generation under matrix-deprived conditions. Thus, our study delineates proline metabolism as an important player in anchorage-independent survival (Figure 4F), suggesting that therapeutic interventions targeting proline metabolism could be developed to prevent metastatic spread.

## 4 Materials and Methods

### 4.1 Cell Types and Culture Conditions

The cell lines used in this study include two cell lines derived from human fibroblasts (received as a kind gift from Dr. Robert A. Weinberg): +ST (read as “plus ST”) cells, stably expressing the following genetic elements: hTERT, oncogenic Ras, simian virus 40 (SV40) large T (LT) and small t (ST) antigens, and –ST (read as “minus ST”) cells stably expressing all of the above genetic elements except SV40 ST antigen (Rangarajan et al., 2004).

Both cell lines were cultured in DMEM (Sigma Aldrich, St Louis, MO, United States) supplemented with 10% fetal bovine serum (FBS; Gibco, Invitrogen) with the addition of antibiotics penicillin and streptomycin, at 37°C and 5% CO_2_. For culture under matrix-deprived conditions, cells were seeded on dishes coated with 1 mg/ml (w/v) poly-2-hydroxyethylmethacrylate (PolyHEMA; Sigma Aldrich) dissolved in absolute ethanol or on dishes coated with 1% agar.

### 4.2 Lentiviral Vector Production

Inducible shRNA against AMPKα1 (Seq #1: V2THS_57696; Seq #2: V2THS_57699) and AMPKα2 (Seq #1: V2THS_57638; Seq #2: V2THS_375,319) were procured from Dharmacon (Pittsburgh, United States). Recombinant lentiviruses were produced by transient co-transfection of pTRIPZ vector encoding either shRNA against AMPKα1 or AMPKα2 under an inducible Tet-on promoter, PMD2. G and psPAX2 in HEK293T cells using turbofect reagent (Thermo Fisher Scientific). Infectious lentiviruses were collected at 36, 48 and 72 h post-transfection, subjected to a freeze-thaw cycle and centrifuged at 500 g for 10 min to remove cellular debris. Concentration of lentiviruses was achieved using lenti-X concentrator (Clontech).

### 4.3 RNAi Experiments

Stable cells expressing shRNA against AMPKα1 or AMPKα2 under a Tet-on inducible promoter were generated in +ST cells by lentiviral transduction. Stable cells were selected by FACS sorting for RFP expression (encoded by the vector) following induction with 5 µg/ml of doxycycline for 48 h. Knockdown was confirmed by qPCR analysis and immunoblotting. Uninduced stable cells were used as control.

### 4.4 Immunoblotting

For immunoblotting, whole cell lysates were prepared using lysis buffer containing 50 mM Tris, 50 mM sodium fluoride, 5 mM sodium pyrophosphate, 1 mM EDTA, 1 mM EGTA, 1% (v/v) triton X-100, DTT, benzamidine and protease inhibitor (Roche) on ice. Protein concentration was estimated using Bradford’s method, and equal quantity of protein (50–75 μg) per lane was resolved by SDS-PAGE after addition of sample dye and boiling for 3 min at 100°C. The proteins were transferred to polyvinylidene difluoride (PVDF) membrane, and probed with appropriate antibodies. The membrane was incubated overnight with primary antibody at 4°C followed by incubation for 2 h with HRP-conjugated secondary antibody at room temperature (RT). Chemiluminescence (using ECL substrate from Thermo Fisher Scientific) was used to visualize protein bands. All western blots are representatives of at least 3 independent experiments. Multi-panel blots were compiled by either reprobing the same blot for successive antibodies, or by loading the same lysate in multiple lanes from a master-mix of prepared sample and independently probing for loading control, *α* Tubulin, in all the repeats.

Primary antibodies used in the study are against pAMPK*α* (T172), pACC (S79), pRaptor (S792), AMPK, ACC, Raptor, AMPKα1, AMPKα2 (Cell Signaling Technology), and *α* Tubulin (Calbiochem). Horseradish peroxidase-conjugated anti-mouse and anti-rabbit antibodies were obtained from the Jackson ImmunoResearch Laboratories.

### 4.5 Pharmacological Compounds

Pharmacological compounds used in cell culture include, AMPK inhibitor, 6-[4-(2-piperidin-1-ylethoxy-phenyl)]-3-pyridin-4-yl-pyrrazolo [1, 5-a]-pyrimidine (compound C; 10 μM), and AMPK activator A-769662 (150 μM) referred to as A76 in figures, from the University of Dundee, Scotland. Dimethyl sulfoxide (DMSO) was used as vehicle control. For experiments involving proline supplementation, the concentration of proline (dissolved in water) used was 200 mg/L (w/v).

### 4.6 RNA Isolation and Real-Time Polymerase Chain Reaction Analysis

Cells grown till 80% confluency were washed twice with phosphate-buffered saline (PBS), and TRIzol (Invitrogen) was added. Total RNA was isolated according to the manufacturer’s instructions. The quantity and quality of each RNA sample were measured spectrophotometrically using NanoDrop 1000 (Thermo Scientific). cDNA synthesis was performed using the SuperScript III RT cDNA synthesis kit, Invitrogen, starting with 2 μg of RNA. cDNA was diluted 5-fold in RNase-free water (Sigma). Real-time PCR was performed in the Realplex machine (Eppendorf) using SYBR Green Supermix (Finnzymes, Thermo Scientific), 2 μl of 1:5 diluted cDNA, and 10 nM primers in a total volume of 10 μl. Samples were amplified under the following conditions: 95°C for 3 min, followed by 40 cycles at 95°C for 30 s, 60°C for 30 s and 72°C for 30 s. PCR was checked for non-specific products by performing a melting curve analysis (65–95°C). Data were analyzed using the Realplex software system (Eppendorf) and were expressed as relative gene expression (fold increase) using the 2^−ΔΔ*Ct*^ method. The stably expressed gene, β2M, was used as an internal loading control. Specific primers used in this study (Supplementary Table S1) were designed using primer 3.0 software.

### 4.7 Nuclear Magnetic Resonance Spectroscopy

#### 4.7.1 Preparation of Cell Lysate

For cells in attached condition: After removing the media, cells were scraped and metabolites were extracted using methanol extraction protocol. The cells were quenched using liquid nitrogen (5 min) to cease all metabolic activities and were scraped in 600 µl of CD_3_OD:D_2_O (4:1) solution. Sonication was performed 5 times with an interval of 1 min on ice between each sonication. The solution was centrifuged for 20 min at 10,000 rpm. The supernatant was stored at −80°C and later NMR experiments were recorded.

For cells in detached condition: The media was removed by centrifuging for 3 min at 3,000 rpm and 600 µl of CD_3_OD:D_2_O (4:1) was added to the cell pellet. The remaining steps were identical to those performed for cells in the attached condition.

#### 4.7.2 NMR Experiments

2D [^13^C, ^1^H] HSQC experiment was recorded on all repeats of all samples at 298 K using Bruker Avance NMR spectrometer at a ^1^H operating frequency at 700 MHz, equipped with a cryoprobe. 4,096 points and 256 points were recorded for direct (^1^H) and indirect (^13^C) dimension with 8 scans. 2D [^13^C, ^1^H] HSQC-TOCSY experiment was recorded for one repeat of +ST cells under the attached condition at 298 K using Bruker Avance NMR spectrometer at a ^1^H operating frequency at 800 MHz, equipped with a cryoprobe. 4,096 points and 256 points were recorded for direct (^1^H) and indirect (^13^C) dimension with 16 scans and 60 ms TOCSY mixing time.

The spectra were processed using Topspin 3.2 and analyzed using CCPN (Mehlen and Puisieux, 2006).

The 2D [^13^C, ^1^H] HSQC spectrum of all 5 repeats were overlayed to check for reproducibility. We found few peaks (Figure 3B, marked in circle) which were exclusively present in the 2D [^13^C, ^1^H] HSQC spectrum of all the repeats of +ST cells. Using 2D [^13^C, ^1^H] HSQC-TOCSY, we found out those peaks belong to the same spin system/metabolite. This spin system was assigned to proline using software PROMEB (Hanahan and Weinberg, 2011).

### 4.8 MTT Assay

MTT [3-(4,5-Dimethylthiazol-2-yl)-2,5-diphenyltetrazolium bromide] assay was used to assess the survival and proliferation of cells. Cellular enzymes reduce tetrazolium dye MTT into insoluble formazan crystals. The crystals were dissolved in DMSO to give a purple coloration, the absorbance of which was measured at 570 nm using 660 nm as the reference wavelength. 6 × 10^3^ cells were seeded per well under matrix-deprived conditions in 96-well ultra-low attachment plate (Corning) and grown for a specified duration of time, after which they were incubated for 4 h in the presence of 1 mg/ml MTT (Sigma Aldrich). Cell pellet was obtained by centrifugation at 3,500 rpm for 10 min; DMSO was added to the pellet and absorbance was recorded.

### 4.9 Luciferase-Based ATP Assay

Cellular ATP levels were estimated using a luciferase-based assay kit (MAK135; Sigma Aldrich) according to the manufacturer’s instructions. Briefly, cells grown under matrix-attached or matrix-deprived conditions were lysed to release ATP. Luciferin in the presence of luciferase and cell lysate (containing ATP) emitted light, and this luminescence was measured. The light intensity is a direct measure of the intracellular ATP concentration.

### 4.10 Methyl Cellulose Colony Formation Assay

Dishes were coated with 0.6% agar (Sigma Aldrich) in DMEM, and 5 × 10^4^ cells admixed with 1.5% methyl cellulose (Sigma Aldrich) containing DMEM with 10% FBS were layered over it. The dishes were maintained in a humidified chamber for 15 days at 37°C and 5% CO_2_.

### 4.11 Statistical Analysis

Statistical analyses were performed using the GraphPad Prism 5.0 software using either Student’s t-test or one-way ANOVA as applicable. All data are presented as mean ± SEM, where *: *p* < 0.05; **: *p* < 0.01; ***: *p* < 0.001.

## Data Availability

The original contributions presented in the study are included in the article/Supplementary Material, further inquiries can be directed to the corresponding author.
