# Supplementary material for: Sustained AMPK Activation and Proline Metabolism Play Critical Roles in the Survival of Matrix-Deprived Transformed Cells
Source: Front Cell Dev Biol. 2021 Nov 15;9:771366. doi: 10.3389/fcell.2021.771366 (PMC8634847; doi:10.3389/fcell.2021.771366)
Supplement: Supplementary file 1 [file Table1.docx]

Supplementary Material

# Supplementary Tables

## Supplementary Table S1

| **Gene** | **Primer Sequence** |
| --- | --- |
| P5CS/ALDH18A1 FW | 5ʹ-ACCTGCAGGGGGTAAATGTTATTA-3ʹ |
| P55CS/ALDH18A1 RV | 5ʹ-GGTTCCAAATGTCACAGACTGC-3ʹ |
| PYCR1 FW | 5ʹ-CATGACCAACACTCCAGTCG-3ʹ |
| PYCR1 RV | 5ʹ-CCTTGGAAGTCCCATCTTCA-3ʹ |
| POX/PRODH FW | 5ʹ-CCACAATGAGGACACAGTGC-3ʹ |
| POX/PRODH RV | 5ʹ-GACAAGTAGGGCAGCACCTC-3ʹ |
| PRKAA1 FW | 5ʹ-GACAGCCGAGAAGCAGA-3ʹ |
| PRKAA1 RV | 5ʹ-AGGATGCCTGAAAAGCTT-3ʹ |
| PRKAA2 FW | 5ʹ-ACCAGCTTGCAGTGGCTT-3ʹ |
| PRKAA2 RV | 5ʹ-CAGTGCATCCAATGGACA-3ʹ |
| β2M FW | 5ʹ-CCTGAATTGCTATGTGTCT-3ʹ |
| β2M RV | 5ʹ-TGATGCTGCTTACATGTCT-3ʹ |

# Supplementary figures

**Supplementary Figure** **1**:

**S1A:** Matrix-deprived (Det) +ST cells treated with either vehicle control (DMSO) or AMPK inhibitor, compound C (CC; 10 μM), for 24 h were harvested for immunoblot analyses for the specified proteins; n=3.

**S1B:** +ST cells stably expressing inducible shRNA against AMPKα1 and AMPKα2 were subjected to 5 µg/mL doxycycline induction for 48 h and harvested for qPCR analysis of the respective AMPKα subunit encoding-gene (PRKAA1 or PRKAA2); uninduced cells were used as control. Graph represents fold changes normalized to housekeeping gene β2M. Error bars represent mean ± SEM; n=3.

**S1C:** +ST cells stably expressing inducible shRNA against AMPKα1 and AMPKα2 were subjected to 5 µg/mL doxycycline induction for 48 h and harvested for immunoblotting for the specified proteins. Uninduced cells were used as control; n=3.

**S1D:** Matrix-deprived (Det) –ST cells treated with either vehicle control (DMSO) or AMPK activator, A-769662 (A76; 150 μM), were harvested for immunoblot analyses for the specified proteins; n=3.

**S1E:** (a) 2D [^13^C, ^1^H] HSQC-TOCSY NMR spectrum of cell lysates from +ST cells under adherent conditions. 2D [^13^C, ^1^H] HSQC-TOCSY helped in identifying spin system of peaks exclusive to +ST cells, which was later assigned to proline using software PROMEB. (b) Zoomed in region of peaks found out to be from same spin system using 2D [^13^C, ^1^H] HSQC-TOCSY NMR spectrum.

**S1F:** Adherent +ST cells treated with AMPK inhibitor compound C (CC; 10 µM) or vehicle control DMSO for 24 h were harvested for qPCR analysis of P5CS/ALDH18A1. Error bar represents mean ± SEM; n=3.
