# Supplementary figures and images for: Sustained AMPK Activation and Proline Metabolism Play Critical Roles in the Survival of Matrix-Deprived Transformed Cells
Source: Front Cell Dev Biol. 2021 Nov 15;9:771366. doi: 10.3389/fcell.2021.771366 (PMC8634847; doi:10.3389/fcell.2021.771366)

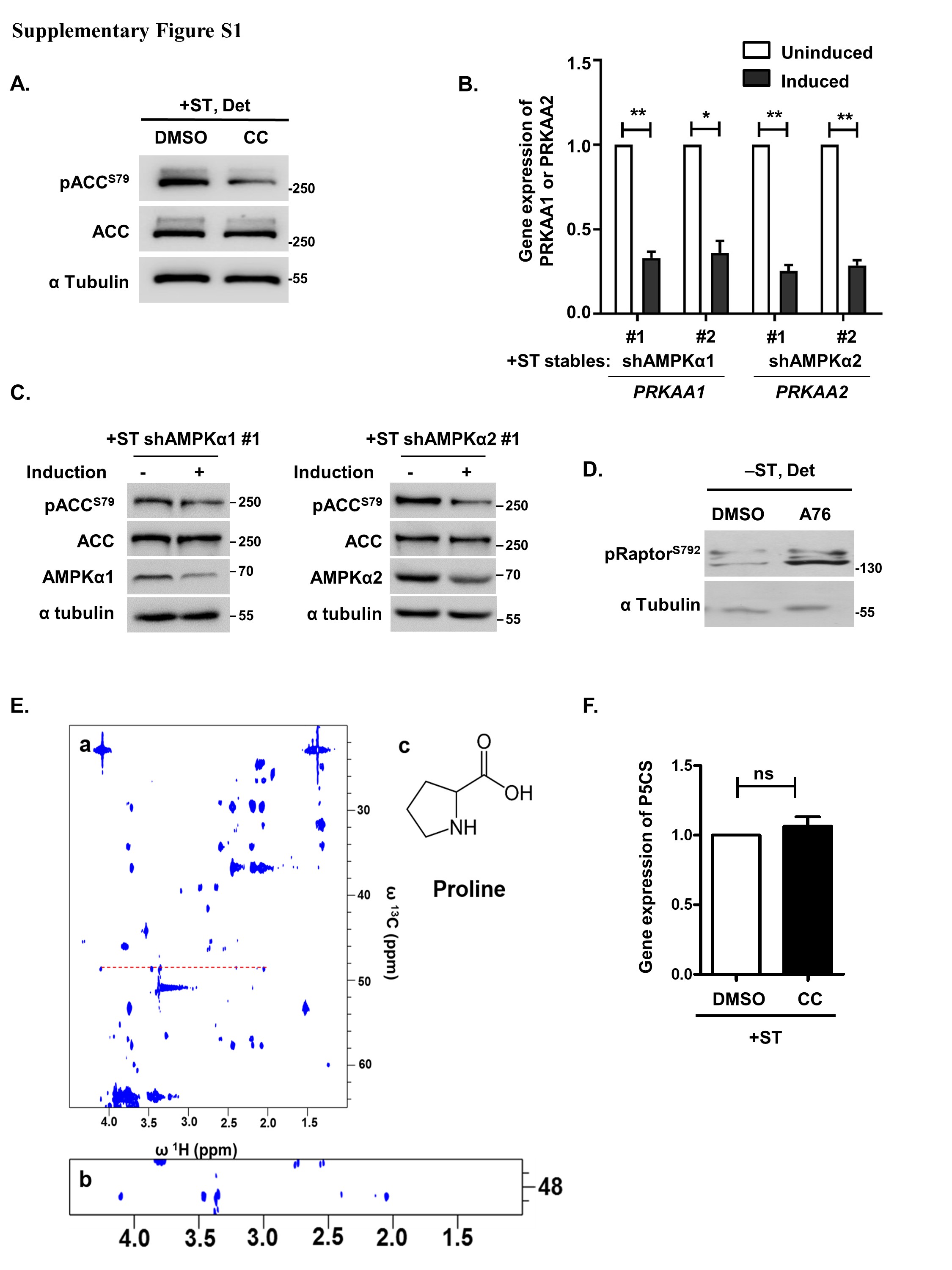

Supplement: Supplementary file 2 [file Image1.JPEG]
